# The avoidance strategy of environmental constraints by an aquatic plant *Potamogeton alpinus* in running waters

**DOI:** 10.1002/ece3.1598

**Published:** 2015-07-22

**Authors:** Alicja Robionek, Krzysztof Banaś, Rafał Chmara, Józef Szmeja

**Affiliations:** Department of Plant Ecology, University of GdańskWita Stwosza 59, PL 80-308, Gdańsk, Poland

**Keywords:** Avoidance strategy, hydrodynamic constraints, phenotypic strategy, trade-off strategy, water flow

## Abstract

Aquatic plants anchored in streams are under pressure from various constraints linked to the water flow and display strategies to prevent their damage or destruction. We assume that the responses of aquatic plants to fast-water flow are a manifestation of a trade-off consisting in either maximizing the resistance to damage (tolerance strategy) in minimizing the hydrodynamic forces (avoidance strategy), or both. Our main hypothesis was that *Potamogeton alpinus* demonstrate the avoidance strategy. We analyzed architecture traits of the modules of this clonal plant from slow- and fast-flowing streams. In fast-flowing waters, the avoidance strategy of *P. alpinus* is reflected by the following: (1) the presence of floating leaves that stabilize the vertical position of the stem and protect the inflorescence against immersion; (2) elongation of submerged leaves (weakens the pressure of water); and (3) shoot diameter reduction and increase in shoot density (weakens the pressure of water, increases shoot elasticity), and by contrast in slow-water flow include the following: (4) the absence of floating leaves in high intensity of light (avoiding unnecessary outlays on a redundant organ); (5) the presence of floating leaves in low intensity of light (avoidance of stress caused by an insufficient assimilation area of submerged leaves).

## Introduction

The aquatic plant species anchored in streams are under the pressure of hydrodynamic forces and other environmental factors related with the flow of water, such as water turbidity, changes in sediment composition, and granulometry (Paterson and Black 1999). The manifested response of plants to such conditions is a phenotypic plasticity that prevents damage to or destruction of their stems, leaves, and other organs. By phenotypic plasticity is meant the ability of an individual organism to alter its morphology and physiology in response to environmental conditions (Schlichting 1986). Effects resulting from the impact of such factors were repeatedly tested in streams, canals, seas, and lakes, both for macroalgae (Molloy and Bolton 1996; Kawamata 2001) and for vascular plants (e.g., Chambers et al. 1991; Schutten and Davy 2000; Bociąg et al. 2009). The hydrodynamic forces, in combination with the remaining environmental conditions, affect the plant metabolism (Nielsen and Sand-Jensen 1993; Titus and Sullivan 2001), their development (Szmeja and Gałka 2008), co-occurrence of species in communities (Chmara et al. 2013), and vegetation structure (Schutten and Davy 2000; Baattrup-Pedersen et al. 2008; Szmeja and Gałka 2013).

The responses of anchored plant species to hydrodynamic forces in water bodies are indicative of a trade-off strategy (Bociąg et al. 2009), which in the case of aquatic plants Puijalon et al. (2011) developed and documented using comprehensive factual material. In light of the trade-off conception, aquatic plant species manifested many peculiar compromises (e.g., Blanchette 1997; Riis and Biggs 2001; Blanchette et al. 2002; Bociąg et al. 2013). In the case of plant species occurring in streams, their responses to hydrodynamic forces could be a manifestation of maximizing the resistance to flow (tolerance strategy) or minimizing the effects of flow (avoidance strategy), or both these reactions manifested simultaneously in various proportions, as variants of a trade-off strategy (Bociąg et al. 2009; Puijalon et al. 2011). The tolerance strategy consists in traits that enable plants to endure adverse conditions and is reflected mostly by maximization of strength of the tissues and maximization of cross-sectional area (Puijalon et al. 2011). The avoidance strategy entails traits that enable plants to resist adverse conditions by preventing the unfavorable effects of these conditions and might be reflected by reconfiguration and/or reduction of the area exposed to flow (Sand-Jensen 2003; Puijalon et al. 2005, 2008), capacity to form dense aggregations (Velasco et al. 2003; Szmeja and Gałka 2008), and changes in shape and structure of leaves, both for submerged and for emerged water plant species (Sand-Jensen and Frost-Christensen 1999; Boeger and Poulson 2003). In this study, we intend to verify the type of strategy of *Potamogeton alpinus* Balb. (alpine pondweed) by analyzing its phenotypic responses in lowland streams.

*Potamogeton alpinus* is a species of aquatic plant that occurs in a substantial part of the Northern Hemisphere, including Eurasia, Greenland, Canada, and northern parts of the United States (Hultén and Fries 1986). It grows in water bodies such as streams, less frequently in ponds, lakes, and marshes. The center of the geographic range of *P. alpinus* in Europe is situated on the Scandinavian Peninsula (Preston 1995), while in northern Poland, where we conducted the research, alpine pondweed occurs rarely, in lowland streams with laminar or turbulent flow. This is a perennial clonal plant anchored via a rhizome (Boedeltje et al. 2005), which produces a large number of seeds (Wiegleb and Todeskino 1985); however, it multiplies primarily vegetatively (Wiegleb et al. 1991; Grace 1993). It is noteworthy that *P. alpinus* has a high phenotypic plasticity, especially such traits as shape and size of submerged leaves and a tendency to produce and develop floating leaves (Kaplan 2002, 2008; Kaplan and Zalewska-Gałosz 2004). According to Kaplan (2002), even within a single genotype such environmental factors as depth, light intensity, or water velocity cause changes in the morphological structure of stems, and submerged and floating leaves, which suggest that these are phenotypic responses.

Our main hypothesis was that *P. alpinus* demonstrate the avoidance strategy. According to Puijalon et al. (2011), macrophytes representing a caulescent unbranched growth form, such as many of the *Potamogeton* species, usually demonstrate the avoidance strategy, not tolerance. The subhypotheses were as follows: (1) environmental conditions in the populations of *P. alpinus* in slow- and fast-flowing streams do not differ significantly; (2) the hydrodynamic constraints affected the architecture of *P. alpinus* in running waters; and (3) mechanical constraints are a major factor, so that in the case of the lack of hydrodynamic constraints, there are no environmental factors affecting the architecture of *P. alpinus*.

## Material and Methods

### Study species

**Figure 1:**
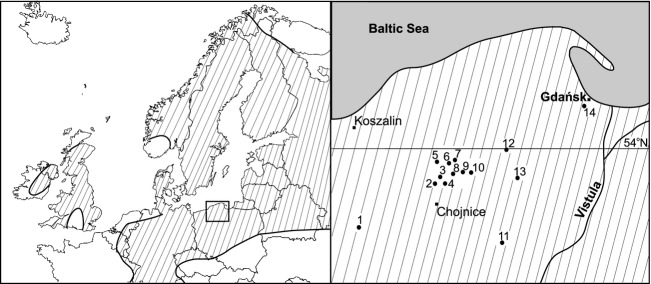
The geographic range of *Potamogeton alpinus* (hatched area, left graph) and distribution of study sites (1–14) in NW Poland (right graph). Study sites (streams): 1 – Komorze, 2 – Ruda, 3 – Chocina, 4 – Czerwona Struga, 5 – Brda near Wilkowo, 6 – Kulawa, 7 – Kłonecznica, 8 – Zbrzyca near Laska, 9 – Zbrzyca near Rolbik, 10 – Zbrzyca near Kaszuba, 11 – Brda near Tuchola, 12 – Graniczna, 13 – Wierzyca, 14 – Oliwa. Box – study area, enlarged on right graph.

*Potamogeton alpinus* is a boreal plant (Meusel et al. 1965; Fig. 1), found up to the polar circle and northward, for example, in the tundra zone (Preston 1995; Bobrov and Chemeris 2009), occurring in well-lit, not very fertile (Boedeltje et al. 2005), shallow, slightly acidic, or alkaline streams (Wiegleb and Todeskino 1983). In the subtemperate climate in the early summer, rhizomes grow leafy stems with roots, which in full summer develop flowers and fruits. In the autumn, aboveground stems decline, and the plant overwinters as rhizomes or turions (Brux et al. 1987; Germ et al. 2002). In north-western and central Europe, *P. alpinus* occurs locally (Baattrup-Pedersen et al. 2008) and in some countries is a vulnerable or endangered element of flora of lowland streams due to eutrophication and/or intoxication (Sand-Jensen et al. 2000; Riis and Sand-Jensen 2001, 2002). The study was performed in north-western Poland (Pomerania region). It is worth mentioning that the southern border of the geographical range of *P. alpinus* in Europe lies in this area, the center of which lies on the Scandinavian Peninsula. Sites in northern Poland are separated from those in Scandinavia by the barrier of the Baltic Sea (Fig. 1).

### Environmental conditions

First, we verified whether there are statistically important differences in environmental conditions between slow- and fast-flowing streams. Environmental conditions in streams were determined on the basis of 18 features of water and sediment, for three measurements per study site, within patches of plants. Water features measured are as follows: 1 – pH, 2 – conductivity (*μ*S/cm), 3 – redox potential (mV), 4 – concentration of total phosphorus (mgTP/dm^3^; spectrophotometrically, for *λ* = 880 nm), 5 – total nitrogen (mg TN/dm^3^; for *λ* = 340 nm), 6 – calcium (mg Ca^2+^/dm^3^), 7 – humic acids (mg/dm^3^), 8 – water flow (m/sec; using the Electromagnetic Flow Meter Valeport M-801), 9 – water color (mg Pt/dm^3^), 10 – light intensity (PAR, %, using LiCOR Li-250 light meter). Sediment features measured are as follows: 11 – pH, 12 – conductivity (*μ*S/cm), 13 – redox potential (mV), 14 – concentration of calcium (mg Ca/g d.w. – dry weight), 15 – organic matter content (%), 16 – mineral matter content (%), 17 – sediment water content (%), 18 – granulometry (fractions: f1 < 0.1, f2 0.1–0.25, f3 0.25–0.5, f4 0.5–1.0, f5 1.0–2.0, f6 > 2.0 mm). The measurements were performed using methods suggested by Wetzel (2001) and Eaton et al. (2005). The significance of differences in environmental conditions between slow- and fast-flowing streams was determined by the Mann–Whitney *U*-test (Sokal and Rohlf 1995).

### Influence of hydrodynamic constraints on plant architecture

The second subhypothesis is to verify whether the hydrodynamic constraints affected the architecture of *P. alpinus* in running waters. The first step was to describe the architecture of the studied plant. During the full growing season (July/August), in the phase of flowering and fruiting of alpine pondweed, we randomly collected 411 plant samples from several meter long sections of 14 lowland streams, including 176 (42.8%) from those with slow-water flow (0.01–0.2 m/sec) and 235 (57.2%) from faster ones (0.2–0.7 m/sec), from a depth of 0.5 m. Stem architecture was determined on the basis of 411 plant samples (flowering modules of this clonal species), that is, repeating structural units consisting of aboveground stem (leaves, shoot, inflorescence) and the stretch of rhizome to the nearest aboveground stem. The object of the study was flowering stems, that is, from the same phase of development. We analyzed 17 traits of stem: 1 – HS (mm; height of shoot), 2 – number of internodes (NI), 3 – length of internode (mm; LI), 4 – number of submerged leaves (NSL), 5 – length of submerged leaf (mm; LSL), 6 – width of submerged leaf (mm; WSL), 7 – area of submerged leaf (mm^2^; LA_SL), 8 – NFL (number of floating leaves), 9 – length of floating leaf (mm; LFL), 10 – width of floating leaf (mm; WFL), 11 – area of floating leaf (mm^2^; LA_FL), 12 – specific leaf area, for both types of leaves (mm^2^ mg^−1^; SLA), 13 – length of rhizome (mm; LR), 14 – biomass of shoot (g d.w.; SB), 15 – biomass of leaves (g d.w.; LB), 16 – biomass of inflorescence (g d.w.; IB), 17 – biomass of rhizome (g d.w.; RB). Afterward, for both variants of the water velocity (i.e., study sites no. 7 and 10), measurements were made of SSD (stem-specific density; mg mm^−3^) and cross-sections of the shoot, and photographs were taken by a stereoscopic microscope. From the digital image, we calculated shoot and stele area (mm^2^), using ImageJ freeware v.1.45 (Abràmoff et al. 2004).

To evaluate the impact of the hydrodynamic constraints on the architecture of *P. alpinus* in running waters, we first determined the significance of differences between the plant traits in two types of watercourses, by the Student's *t*-test or Mann–Whitney *U*-test (Sokal and Rohlf 1995). Afterward, we estimated the influence of 18 environmental factors on 17 plant traits, by the method of PCA (principal components analysis) and RDA (redundancy analysis; ter Braak and Šmilauer 2002), performed by the computer program Canoco 4.5. Preliminary analysis showed that the data obtained were linear in structure (length of gradient 0.4 SD, Detrended Correspondence Analysis), thereby calling for the use of RDA. Statistical significance was tested using the Monte Carlo permutation test (ter Braak and Šmilauer 2002). Additionally, for five architecture traits (i.e., height and biomass of shoot, length of submerged and floating leaf, SLA), we analyzed the variation traits of stems by NMDS (nonmetric multidimensional scaling; Kruskal 1964; Clarke 1993) and one-way analysis of similarities, for Bray–Curtis similarities (Bray and Curtis 1957). Thereafter, we used the SIMPER (similarity percentages). The NMDS and SIMPER analyses used PAST ver. 2.03 (Hammer et al. 2001).

### Are mechanical constraints a major factor?

The last subhypothesis was that in the case of the lack of hydrodynamic constraints, there are no environmental factors affecting the architecture of *P. alpinus*. In order to verify this hypothesis, we repeated the previous steps (included in the testing of the second sub-hypothesis) in the case of the lack of hydrodynamic constraints, that is, in slow-flowing streams.

## Results

### Environmental conditions

Water in streams has a pH 6.8–8.7, conductivity 251 ± 51 *μ*S/cm, and redox potential 83 ± 90 mV and is calcium rich, humic acid-poor and slightly colored (Table S1). The sediment has a pH 6.3–7.7, conductivity 196 ± 139 *μ*S/cm, and redox potential −79 ± 89 mV, is rich in mineral content, is poorly hydrated, contains 15.5 ± 31.5 mg Ca/g d.w., and is grained (with the dominant fraction of 0.25–0.50 mm). The flow velocity is very diverse (0.01–0.7 m/sec). Environmental conditions between slow-flowing (variant of the water flow – A: <0.2 m/sec; sites 1, 4, 7–9, 14, see Fig. 1) and fast-flowing streams (variant of the water flow B: 0.2–0.7 m/sec; sites 2, 3, 5, 6, 10, 11, 12) do not differ in terms of most of the analyzed features, that is, in water: the concentration of TN (*U* = 198, *P* > 0.5), TP (*U* = 192, *P* > 0.5), calcium (*U* = 159, *P* > 0.1), conductivity (*U* = 160, *P* > 0.1), redox potential (*U* = 188, *P* > 0.1), humic acids (*U* = 187, *P* > 0.1), and color of water (*U* = 165, *P* > 0.1); in sediment: calcium concentration (*U* = 211, *P* > 0.5), redox potential (*U* = 160, *P* = 0.1), and granulometry (*P* > 0.1); and light intensity (*U* = 16, *P* > 0.5). Highly significant differences were found only for sediment water content (A: 39.3 ± 24.3%, B: 20.5 ± 6.5%; *U* = 56, *P* < 0.001) and mineral matter content (A: 95.0 ± 4.3%, B: 99.0 ± 0.9%; *U* = 69, *P* < 0.001), but these features are directly dependent on water flow. Among the slow streams (A), there are two types of sites, significantly different in terms of light intensity: well illuminated (PAR 82.7 ± 13.5%) or shaded (PAR 41.2 ± 5.0%; *U* = 0.0, *P* < 0.001).

### Influence of hydrodynamic constraints on plant architecture

**Figure 2:**
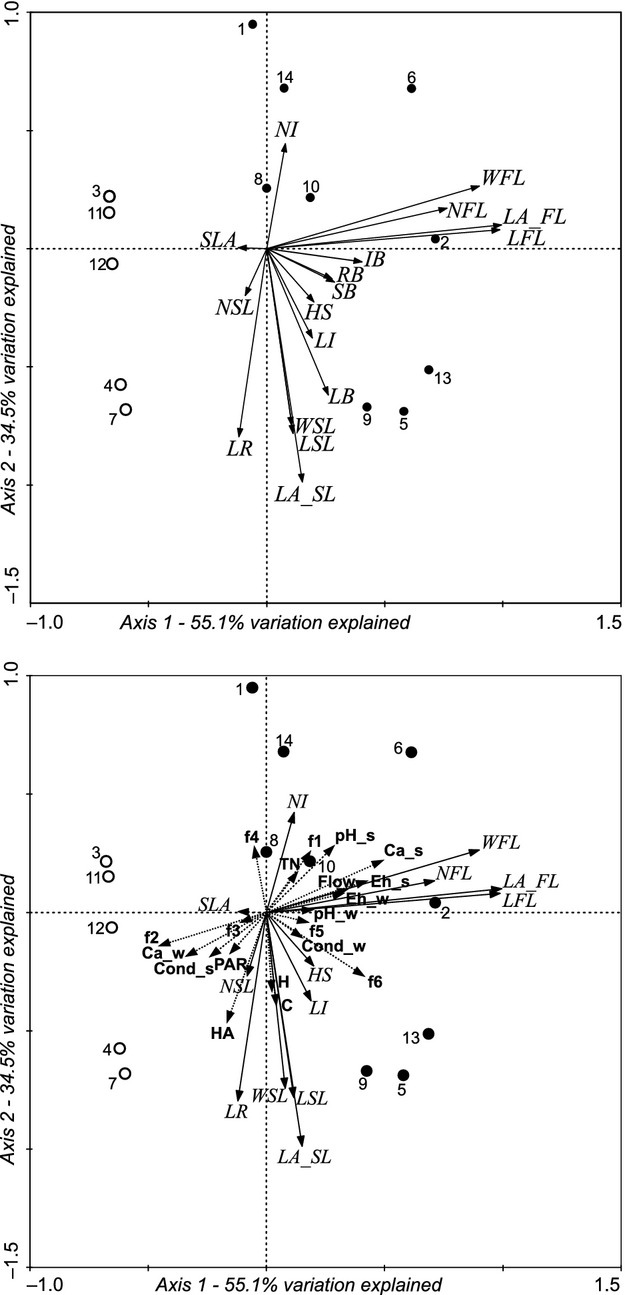
Principal components analysis (upper graph) and redundancy analysis (lower graph) diagrams of *Potamogeton alpinus* traits and environmental factors in streams (1–14), where black dots – stems with floating leaves; white dots – without such leaves. Abbreviations. 1–14: study sites. Architecture traits: HS – height of shoot; NI – number of internodes; LI – length of internode; NSL – number of submerged leaves; LSL – length of submerged leaf; WSL – width of submerged leaf; LA_SL – area of submerged leaf; NFL – number of floating leaves; LFL – length of floating leaf; WFL – width of floating leaf; LA_FL – area of floating leaf; SLA – specific leaf area; LR – length of rhizome; SB – biomass of shoot; LB – biomass of leaf; IB – biomass of inflorescence; RB – biomass of rhizome; water (_w) and sediment (_s) features: Cond – conductivity, Eh – redox potential, Ca – calcium, TN – total nitrogen, HA – humic acids, C – color, SWC – sediment water content, f1–f6 – granulometry of sediment, PAR – photosynthetically active radiation.

There are two types of flowering stems (Fig. 2): the first with floating and submerged leaves (upper right quadrant of the PCA diagram); the second with only submerged leaves (lower part of the PCA diagram). Distinguishing features of the first type of stem architecture are number, width, and area of floating leaves, whereas for the second type, length, width, area of submerged leaves, and biomass of leaves. The occurrence of stems with floating leaves might depend on several environmental factors, including flow velocity, as well as calcium concentration in the water and sediment redox potential (RDA diagram, Fig. 2). However, chemical factors weakly differentiate environmental conditions between the water flow variants (see Table S1).

**Figure 3:**
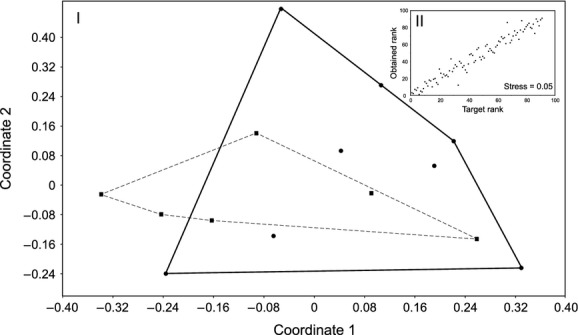
Dissimilarity of stems in slow-water flow (dashed line) and fast-water flow (solid line; panel I), using nonmetric multidimensional scaling. Shepard plot – panel II.

**Figure 4:**
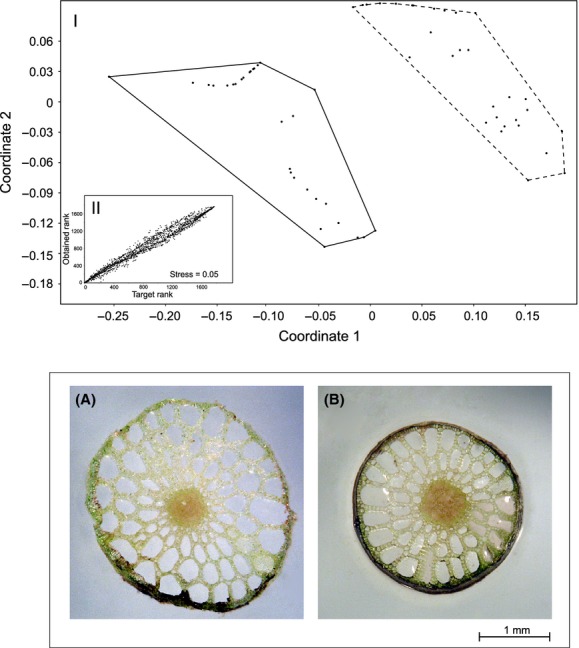
Dissimilarity of stems in slow-water flow (A, dashed line, site no 7) and fast-water flow (B, solid line, site no 10), using nonmetric multidimensional scaling (upper part, panel I), Shepard plot (panel II), and cross-section distinction of the shoots (lower part).

In fast-flowing waters (variant B), in comparison to slow flowing (A), there is higher variance of stems’ features (Fig. 3). The greatest differences were visible in the SLA index (49.4% dissimilarity) and height of shoot (49.2%). Stems in variant B, in comparison to A, differ in the majority of compared traits (Table 1). In high velocity (B), shoots are higher with longer internodes, submerged leaves are longer and more numerous, floating leaves are longer, more numerous, have a higher area, the SLA index (taking into account both types of leaves) is higher, the rhizome is shorter and lighter, and biomass of inflorescence is smaller. In addition, in variant B shoots are thinner (A: 1.60 ± 0.16 mm, B: 1.26 ± 0.25 mm; *Z* = 5.0, *P* < 0.001), their density is higher (A: 0.01 ± 0.003 mg/mm^3^, B: 0.022 ± 0.006 mg/mm^3^; *Z* = −6.3, *P* < 0.001), and also there is greater participation of stele in the cross-section of the stem (A: 2.50 ± 0.61%, B: 6.84 ± 1.03%; t = 15.4, df = 34, *P* < 0.001; Fig. 4). Statistically important dissimilarities in the architecture of stems from selected stands of A and B variants are affected mainly by SLA (49.4% dissimilarity) and height of shoot (42.8%).

**Table 1:** Stems in slowly (A; <0.2 m/sec) and faster flowing (B; 0.2–0.7 m/sec) waters.

			*U*-test	
Plant trait	A	B	*Z*	*P*
Length of submerged leaf [mm]	106.1 ± 27.4	125.4 ± 27.8	3.8	<0.001
Length of floating leaf [mm]	60.9 ± 10.7	92.1 ± 25.6	6.6	<0.001
Area of floating leaf [mm^2^]	918.0 ± 318.4	1238.9 ± 486.2	−4.0	<0.001
Inflorescence biomass [g d.w.]	0.05 ± 0.05	0.02 ± 0.03	4.2	<0.001
Length of rhizome [mm]	117.5 ± 60.6	97.9 ± 38.2	3.0	<0.01
Specific leaf area [mm^2^ mg^−1^]	443.9 ± 362.6	563.0 ± 387.2	−2.7	<0.01
Number of floating leaves	3.7 ± 1.8	2.7 ± 1.3	3.4	<0.01
Height of shoot [mm]	384.3 ± 143.9	489.8 ± 304.5	−2.1	<0.05
Length of internodes [mm]	24.6 ± 7.7	30.6 ± 16.1	−3.0	<0.05
Number of submerged leaves	11.6 ± 3.5	12.3 ± 2.9	−2.0	<0.05
Rhizome biomass [g d.w.]	0.08 ± 0.06	0.06 ± 0.05	2.3	<0.05
Number of internodes	16.1 ± 4.4	15.7 ± 3.6	0.3	>0.05
Width of submerged leaf [mm]	17.9 ± 3.9	17.1 ± 4.1	1.4	>0.05
Area of submerged leaf [mm^2^]	1618.5 ± 685.4	1758.3 ± 608.9	−1.2	>0.05
Shoot biomass [g d.w.]	0.13 ± 0.09	0.14 ± 0.10	−1.1	>0.05
Leaves biomass [g d.w.]	0.33 ± 0.19	0.31 ± 0.16	0.8	>0.05
Width of floating leaf [mm]	18.6 ± 4.0	17.1 ± 3.5	1.6	>0.05
Number of samples	176	235		

Explanations: d.w. – dry weight.

### Are mechanical constraints a major factor?

**Figure 5:**
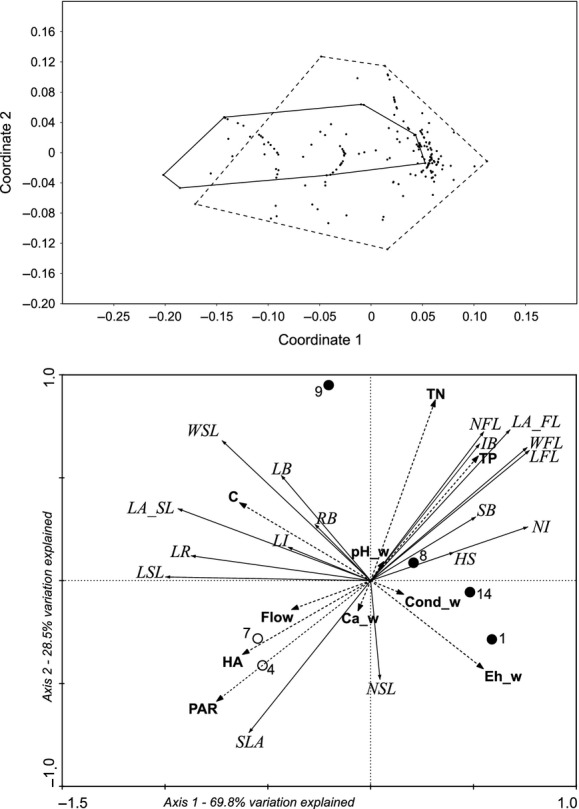
Dissimilarity of stems from illuminated (solid line) and shaded (dashed line) slow streams, using nonmetric multidimensional scaling (upper graph) and redundancy analysis diagram (lower graph) of environmental factors and plant traits in illuminated (white dots) and shaded (black dots) slow streams. Abbreviations. 1–14 – study sites, HS – height of shoot; NI – number of internodes; LI – length of internodes; NSL – number of submerged leaves; LSL – length of submerged leaves; WSL – width of submerged leaves; LA_SL – leaf area of submerged leaves; NFL – number of floating leaves; LFL – length of floating leaves; WFL – width of floating leaves; LA_FL – leaf area of floating leaves; SLA – specific leaf area; LR – length of rhizome; SB – biomass of shoot; LB – biomass of leaf; IB – biomass of inflorescence; RB – biomass of rhizome; water (_w) features: Cond – conductivity, Eh – redox potential, Ca – calcium, TN – total nitrogen, TP – total phosphorus, HA – humic acids, C – color, PAR – photosynthetically active radiation.

Stems with floating leaves occur in fast-flowing streams, as well as in slowly flowing and shaded waters (<50% PAR light intensity); however, the ones without floating leaves occur in slow and illuminated waters (>75% PAR). Stems from illuminated and shaded slow streams are statistically dissimilar as regards the SLA index (68.3%) and height of shoot (22.2%, Fig. 5, upper graph). Light intensity is the main environmental factor which determines the presence or absence of floating leaves (RDA diagram, Fig. 5). From our multiple and annually repeated measurements of light intensity it follows that they are created in the shadows (in the forest streams), while not formed under conditions of good lighting (in nonforest streams).

## Discussion

The dominant factor shaping the ecology of aquatic systems is water flow (Paterson and Black 1999). Water movement, such as flow or wave action, causes erosion of the substrate or accumulation of matter which is sorted into various granulation fractions (Paterson and Black 1999; Vermaat et al. 2000). The only significant differences in environmental factors between slow and fast streams were found for sediment water content and mineral/organic matter content, but these features are directly dependent on water flow. In slow-flowing watercourses, sediment is organic and fine grained, while in fast-flowing watercourses, it is mineral and coarse grained.

The resistance of water plants to hydrodynamic forces and to other environmental conditions depends not only on such factors, but also on plasticity of the species. This is confirmed by the results of numerous studies, including Biehle et al. (1998), who studied *Fontinalis antipyretica*, Boeger and Poulson (2003), who examined *Veronica anagalis-aquatica*, or Miler et al. (2012), who compared plant–flow interactions of various species. What is more, responses to the same underlying environmental influences differed at least partially among coexisting species (Freschet et al. 2013). The plant species exposed to environmental constraints display various plastic responses in their morphological, anatomical, physiological, or reproductive traits that can reduce the detrimental effects (Szmeja 1987; Sultan 2000, 2003). The phenotypic responses of water plants to fast-water flow are a manifestation of the trade-off, consisting in either maximizing the resistance to damage (tolerance strategy) or minimizing the hydrodynamic forces (avoidance strategy), or both (Puijalon et al. 2011).

The manifestation of the avoidance strategy of *Potamogeton alpinus* is enhanced stabilization of the stem due to the presence of floating leaves, lower susceptibility of submerged leaves to mechanical damage due to their elongation, reduction in the pressure of water on the shoot of smaller diameter, and strengthening of the shoot by changes in its internal structure. The example of such a strategy can also be the reconfiguration of the stem and/or reduction of the area exposed to flow (Sand-Jensen 2003; Puijalon et al. 2005, 2008), or even the capacity to form dense aggregations (Velasco et al. 2003; Szmeja and Gałka 2008). The flowering stem with floating leaves is a typical architectural form of *P. alpinus*, occurring in fast-flowing lowland streams. The floating leaves absorb CO_2_ and other gases from the air (Nielsen and Sand-Jensen 1993; Titus and Sullivan 2001), but also they stabilize the stem and hold it vertically as well as hold the inflorescence above the water surface, which prevents the immersion of flowers and enables pollination. We link this type of reaction with the avoidance of mechanical damage to or destruction of the stem. Another manifestation of the avoidance strategy is elongation of the shoot. The flowering stem of *P. alpinus* that occurs in fast flow is not vertical (it is inclined); consequently, when it grows up to the water surface, it is longer than the plant in slow water flow. The reason for the elongation of the stem could also be a deficit of light in the turbulent flow (Boedeltje et al. 2005), as well as the placement of photosynthetically active tissues closer to the water surface (Pilon and Santamaría 2002; Cronin and Lodge 2003). It should be pointed out that water plants are known which reduce the height of their stems in fast flow conditions (Idestam-Almquist and Kautsky 1995; Strand and Weisner 2001).

In fast-flowing waters, submerged leaves are more elongated and as a result, they are less exposed to this destruction. Smaller, thinner, and/or more elongated leaves in the submerged plants at high velocity have decreased hydraulic resistance, thereby reducing mechanical damage to the leaf by fast-flowing water (Schutten and Davy 2000; Boeger and Poulson 2003) and avoiding consequences of hydrodynamic disturbances. Moreover, characteristic changes in the shape and structure of leaves were repeatedly observed, both for submerged and for emerged water plant species (Sand-Jensen and Frost-Christensen 1999; Boeger and Poulson 2003), which can be associated with the avoidance strategy. It is worth noting that leaf strategies are conserved during the diversification of vascular plants, especially herbaceous species (Flores et al. 2014).

Another expression of avoidance strategy is the smaller diameter of the stem in fast-flowing waters, which is a response to the flow as a mechanical constraint (e.g., Boeger and Poulson 2003). In the case of anchored aquatic plants occurring in fast-running waters or in shallow lakes with strong wave action, it was found that some species reduce the height of shoots and length of internodes (Chambers et al. 1991) or elasticize the construction of stems (Bociąg et al. 2009), thereby mitigating the pressure of hydrodynamic forces.

Another manifestation of the avoidance strategy of *P. alpinus* is strengthening of the shoot (see Fig. 4). In fast-flowing watercourses, its density (SSD) is higher than in slow flow. Moreover, in fast flow there is greater participation of stele in the cross-section of the stem than in slowly flowing waters. Bociąg et al. (2009) found that the stem of *Potamogeton natans* is more resistant to stretching (15.6 ± 4.7 N) than *P. pectinatus* (3.3 ± 1.0 N), *Batrachium fluitans* (2.6 ± 0.8 N) and *Chara fragilis* (0.6 ± 0.3 N); the latter is typical for standing waters. The ultimate bending moment of *P. natans* stems from lakes (9.7•10^−3^ ± 4.2•10^−3^ Nm) is higher than that from streams (2.1•10^−3^ ± 1.00•10^−3^ Nm). It means that stems from watercourses are more elastic and therefore less prone to damage by stretching forces. We combine this type of reaction with the avoidance strategy, as well as all the other responses found in populations of *P. alpinus*. It is worth noting that the resistance of stems to bending and breaking depends also on the arrangement of strengthening tissue inside it (Bociąg et al. 2009). It can be located centrally (model of a flexible rod) or spherically (nonelastic pipe model). In *P. alpinus*, changes in the internal structure of the shoot consist of an increase in SSD and the participation of stele in the cross-section of the stem, resulting in fast-water flow resistance of the stem being higher (Molloy and Bolton 1996; Kawamata 2001). On the other hand, this strategy may be expressed by an increase in the allocation of biomass of exposed organs, which leads to them obtaining greater resistance to damage (Szmeja and Bazydło 2005). It was found that, for example, the biomass of the anchoring system increased in the populations of *P. pectinatus* and *P. perfoliatus* occurring in water flow and in shallow lakes disturbed by wave action (Szmeja and Gałka 2008).

When the hydrodynamic constraints are absent, *P. alpinus* reacts to insufficient light intensity; thus, the hypothesis of the absence of additional factors should be rejected. The reflection of avoiding too little illumination is the presence of floating leaves in slow and shaded streams, where the floating leaves mitigate the effects of insufficient light intensity, as another environmental constraint. Thus, in this case, the presence of floating leaves is a manifestation of avoidance strategies regarding too little light intensity. Decreasing light availability in many cases causes an increase in specific leaf area (Rijkers et al. 2000; Evans and Poorter 2001), that is, there is an increase in the area of light capture for a constant amount of resource invested. Flowering stems without floating leaves are only present in well-illuminated slow streams, namely in conditions in which such leaves are unnecessary. Under such conditions, lack of floating leaves is a manifestation of the avoidance of unnecessary expenditure of mass (and energy) on the redundant organ.

## Conclusions

In fast-water flow, the avoidance strategy of *P. alpinus* is reflected by the following: (1) the presence of floating leaves that stabilize the vertical position of the stem and protect the inflorescence against immersion; (2) elongation of submerged leaves (weakens the pressure of water); (3) shoot diameter reduction and increase in shoot density (weakens the pressure of water and increases shoot elasticity), and by contrast in slow water flow include the following: (4) in conditions of highlight intensity, floating leaves are not formed (avoiding unnecessary outlays on a redundant organ); (5) in low intensity of light, floating leaves are present (avoidance of stress caused by an insufficient assimilation area of submerged leaves).

## Supporting Information

**Figure S1.** *Potamogeton alpinus* in Zbrzyca River, Poland.

**Table S1.** Water and sediment characteristics on sites (1-14) with slowly* (<0.2 m/sec) and faster (0.2–0.7 m/sec) flowing waters.
