# Supplementary figures and images for: The avoidance strategy of environmental constraints by an aquatic plant Potamogeton alpinus in running waters
Source: Ecol Evol. 2015 Jul 22;5(16):3327–37. doi: 10.1002/ece3.1598 (PMC4569029; doi:10.1002/ece3.1598)

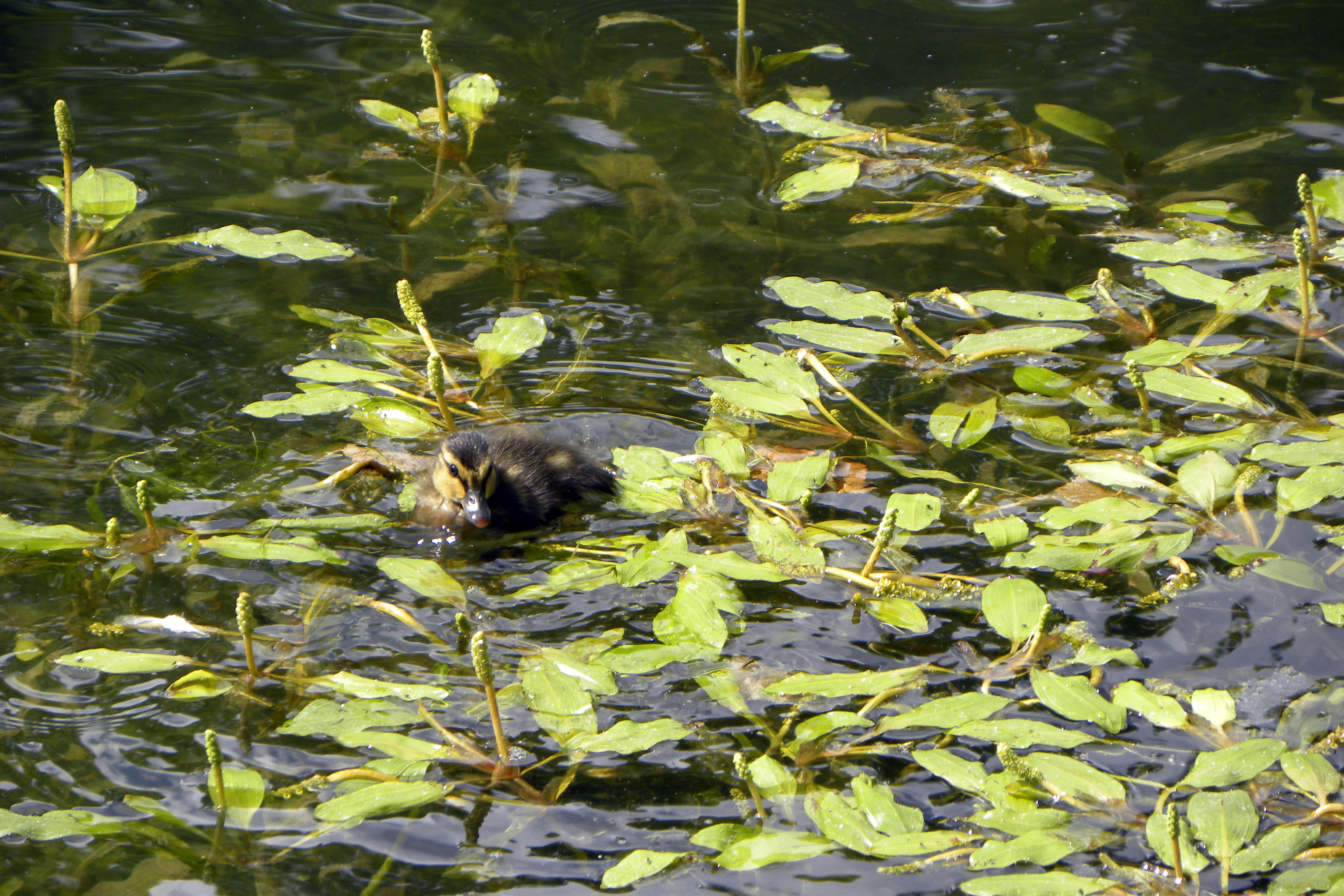

Supplement: Supplementary file 1 — Figure S1. Potamogeton alpinus in Zbrzyca River, Poland. [file ece30005-3327-sd1.tif]
