# Supplementary material for: The avoidance strategy of environmental constraints by an aquatic plant Potamogeton alpinus in running waters
Source: Ecol Evol. 2015 Jul 22;5(16):3327–37. doi: 10.1002/ece3.1598 (PMC4569029; doi:10.1002/ece3.1598)
Supplement: Supplementary file 2 — Table S1. Water and sediment characteristics on sites (1-14) with slowly* (<0.2 m/sec) and faster (0.2–0.7 m/sec) flowing waters. [file ece30005-3327-sd2.doc]

Table S1. Water and sediment characteristics on sites (1-14) with slowly* (<0.2 m/s) and faster (0.2 – 0.7 m/s) flowing waters.

Explanations: Flow (m/s), Cond. – conductivity (µS/cm), Eh – redox potential (mV), Ca – calcium (mg/dm^3^), TN – total nitrogen (mg/dm^3^), TP – total phosphorus (mg/dm^3^), HA – humic acid (mg/dm^3^), C – colour (mg Pt/dm^3^), PAR – light intensity (%), OM – organic matter (%), MM – mineral matter content (%), H – hydration (%), s.d. – standard deviation, me – median; for three measurements of feature characteristics per site.

| Study site | Water | | | | | | | | | | Sediment | | | | | | |
| --- | --- | --- | --- | --- | --- | --- | --- | --- | --- | --- | --- | --- | --- | --- | --- | --- | --- |
|  | Flow | pH (me) | Cond. | Eh | Ca | TN | TP | HA | C | PAR | pH (me) | Cond. | Eh | Ca | OM | MM | H |
| 1* | 0.01±0.00 | 6.8 | 173±13 | 256±18 | 58.1±0.2 | 1.7±0.4 | 0.3±0.0 | 1.2±0.1 | 5±1 | 44.4±3.1 | 7.1 | 202±66 | -79±13 | 21.3±0.6 | 2.7±0.4 | 97.3±0.4 | 28.9±2.2 |
| 7* | 0.05±0.01 | 7.8 | 185±19 | 63±16 | 46.0±0.2 | 1.5±0.3 | 0.1±0.0 | 2.6±0.2 | 20±2 | 70.7±2.7 | 7.1 | 95±48 | -50±20 | 4.2±0.4 | 1.2±0.2 | 98.8±0.2 | 28.8±3.6 |
| 9* | 0.06±0.01 | 7.5 | 213±12 | 27±12 | 56.4±0.4 | 2.3±0.7 | 0.4±0.1 | 2.0±0.1 | 37±3 | 38.0±0.6 | 7.1 | 367±61 | -42±25 | 0.8±0.1 | 11.7±0.5 | 88.3±0.5 | 90.8±2.9 |
| 14* | 0.06±0.01 | 8.7 | 332±24 | 63±19 | 56.6±0.8 | 1.7±0.3 | 0.3±0.1 | 2.6±0.1 | 25±2 | 36.8±1.7 | 7.2 | 196±18 | -79±17 | 6.7±0.6 | 9.5±1.6 | 90.5±1.6 | 28.5±1.8 |
| 4* | 0.16±0.03 | 7.3 | 270±23 | 62±23 | 67.3±1.1 | 1.0±0.1 | 0.1±0.0 | 5.5±0.5 | 40±6 | 94.9±3.3 | 6.3 | 519±65 | -86±4 | 3.0±0.2 | 4.4±1.1 | 95.6±1.1 | 38.4±1.6 |
| 8* | 0.16±0.06 | 7.5 | 255±28 | 38±1 | 45.2±1.7 | 1.5±0.8 | 0.5±0.2 | 2.5±1.2 | 21±1 | 45.9±5.1 | 7.4 | 175±84 | -87±188 | 8.9±3.2 | 0.5±0.3 | 99.5±0.3 | 20.6±4.2 |
| mean | 0.08 | - | 239.2 | 84.9 | 55 | 1.6 | 0.3 | 2.7 | 24.6 | 55.1 | - | 259.1 | -70.4 | 7.5 | 5 | 95 | 39.3 |
| s.d. | 0.06 | - | 57.6 | 80.4 | 7.8 | 0.5 | 0.1 | 1.4 | 11.9 | 23.0 | - | 148.5 | 67.5 | 7 | 4.3 | 4.3 | 24.3 |
| min. | 0.01 | 6.8 | 173 | 27 | 44 | 0.6 | 0.1 | 1.1 | 5 | 36.8 | 6.3 | 79 | -222 | 1 | 0.3 | 88.3 | 18 |
| max. | 0.22 | 8.7 | 332 | 268 | 67 | 2.3 | 0.7 | 5.5 | 40 | 94.9 | 7.4 | 519 | 128 | 22 | 11.7 | 99.7 | 91 |
| me | 0.06 | 7.5 | 218 | 63 | 56 | 1.7 | 0.3 | 2.6 | 23 | 45.1 | 7.1 | 202 | -79 | 5 | 3.6 | 96.4 | 29 |
|  |  |  |  |  |  |  |  |  |  |  |  |  |  |  |  |  |  |
| 5 | 0.2±0.00 | 7.9 | 257±8 | 79±11 | 57.8±1.0 | 2.2±0.2 | 0.4±0.0 | 1.7±0.1 | 19±1 | 58.3±4.6 | 6.9 | 119±79 | -93±10 | 51.5±5.0 | 0.8±0.2 | 99.2±0.2 | 15.3±3.7 |
| 12 | 0.3±0.07 | 7.7 | 269±21 | 15±36 | 61.3±4.3 | 2.9±2.2 | 0.4±0.3 | 3.8±2.8 | 13±3 | 64.6±1.4 | 7.2 | 349±62 | -147±94 | 1.7±0.6 | 1.0±0.5 | 99.0±0.5 | 27.1±9.0 |
| 13 | 0.3±0.05 | 7.7 | 340±47 | 318±6 | 62.1±0.3 | 0.9±0.1 | 0.2±0.1 | 3.4±0.1 | 29±6 | 54.0±0.4 | 7.2 | 114±133 | -135±32 | 16.7±9.2 | 0.5±0.1 | 99.5±0.1 | 20.1±3.8 |
| 11 | 0.3±0.02 | 8.0 | 220±12 | 48±15 | 77.0±0.3 | 1.6±0.5 | 0.3±0.1 | 1.1±0.1 | 15±5 | 49.9±2.4 | 7.7 | 151±12 | -183±47 | 3.7±0.3 | 1.9±1.4 | 98.1±1.4 | 27.3±7.1 |
| 3 | 0.4±0.06 | 7.6 | 290±23 | 43±13 | 58.6±0.0 | 1.1±0.2 | 0.3±0.1 | 2.0±0.6 | 26±8 | 50.2±3.4 | 7.5 | 71±50 | -162±32 | 2.9±0.9 | 0.7±0.3 | 99.3±0.3 | 19.1±2.0 |
| 6 | 0.5±0.09 | 7.8 | 220±35 | 75±84 | 41.7±1.2 | 1.5±0.9 | 0.2±0.2 | 1.2±0.2 | 7±1 | 44.8±1.6 | 7.3 | 248±105 | 16±107 | 89.1±8.4 | 2.3±1.5 | 97.7±1.5 | 21.9±3.7 |
| 2 | 0.5±0.01 | 7.7 | 272±25 | 62±9 | 59.4±1.2 | 2.6±0.2 | 0.1±0.0 | 3.5±0.2 | 20±3 | 42.4±1.2 | 7.1 | 84±12 | -75±16 | 2.9±0.3 | 0.4±0.1 | 99.5±0.1 | 21.6±2.1 |
| 10 | 0.7±0.02 | 7.5 | 214±19 | 9±14 | 42.2±0.4 | 1.7±0.2 | 0.2±0.1 | 2.1±0.2 | 55±4 | 68.8±6.0 | 7.8 | 60±11 | 95±28 | 3.0±0.3 | 0.2±0.1 | 99.8±0.1 | 11.4±0.9 |
| mean | 0.39 | - | 260.4 | 81.3 | 57.5 | 1.8 | 0.3 | 2.4 | 22.8 | 54.1 | - | 149.6 | -85.5 | 21.5 | 1 | 99 | 20.5 |
| s.d. | 0.15 | - | 45.5 | 98.7 | 11 | 1 | 0.2 | 1.3 | 14.5 | 9.2 | - | 112.8 | 102.8 | 40.4 | 1 | 1 | 6.5 |
| min. | 0.22 | 7.5 | 181 | -26 | 41 | 0.5 | 0.1 | 0.6 | 5 | 42.4 | 6.7 | 25 | -243 | 1 | 0.2 | 96.3 | 11 |
| max. | 0.67 | 8.1 | 367 | 322 | 77 | 5.3 | 0.6 | 6.1 | 55 | 68.8 | 7.8 | 402 | 135 | 188 | 3.7 | 99.8 | 37 |
| me | 0.34 | 7.7 | 260 | 51 | 59 | 1.7 | 0.2 | 2.1 | 20 | 52.1 | 7.3 | 107 | -94 | 3 | 0.7 | 99.3 | 21 |
